# Assessment and Treatment of Target Behavior Maintained by Social Avoidance

**DOI:** 10.3390/bs14100957

**Published:** 2024-10-16

**Authors:** Sarah K. Slocum, Emily Gottlieb, Mindy Scheithauer, Colin Muething

**Affiliations:** 1 Marcus Autism Center, 1920 Briarcliff Road, Atlanta, GA 30329, USA; 2 School of Medicine, Emory University, Atlanta, GA 30322, USA

**Keywords:** functional analysis, functional communication training, social avoidance, social conditioning, target behavior

## Abstract

Past research has identified that some individuals with intellectual and developmental disabilities who engage in target behavior (e.g., aggression, self-injury) maintained by negative reinforcement engage in the behavior to escape or avoid social interaction specifically (i.e., social avoidance). However, assessment and treatment strategies for this function are understudied when compared to target behavior maintained by other forms of negative reinforcement. The current study builds on this limited research and demonstrates (a) a replication of functional analysis conditions and a negative reinforcement latency assessment to identify the specific types of social interaction that evoke target behavior, and (b) an intervention that includes stimulus fading, social conditioning, and differential reinforcement for five participants with autism spectrum disorder. Participant target behavior decreased within the intervention phase for four out of five participants. The implications of strategies to guide the use of antecedent-based treatment strategies are discussed for target behavior maintained by social avoidance.

## 1. Assessment and Treatment of Target Behavior Maintained by Social Avoidance

Research has consistently demonstrated the efficacy of interventions based on the results of functional analyses [1] for behavior targeted for reduction, hence termed target behavior. (Challenging behavior and problem behavior are predominant terms used to reference behavior targeted for reduction. Due to criticisms of these terms, we have elected to use *behavior targeted for reduction* and *target behavior*. In this context, these terms denote topographies of behavior that either (a) present a safety risk to the individual or others, or (b) interfere with the individual’s participation in community settings). Some topographies of target behavior include aggression, self-injury, and property destruction [2,3]. In this treatment model, therapists implement functional analyses to determine the function (i.e., the reinforcer maintaining the individual’s target behavior) by systematically manipulating antecedents and consequences commonly related to target behavior [1]. Common conditions include tests for target behavior maintained by access to attention, tangible items, and escape from non-preferred tasks or activities [4]. In each of these test conditions, the therapist restricts the reinforcer evaluated at the start of a session and provides the reinforcer for a brief time contingent on target behavior. This is repeated throughout the course of a session (often 5 or 10 min). For example, in a tangible test condition, a therapist may restrict a preferred item and then deliver it for 30 s contingent on target behavior. In an escape condition, a therapist may present tasks (e.g., homework), often using a least-to-most prompting sequence, and allow the child a 30 s break contingent on target behavior. Therapists record target behavior during these test conditions and compare those levels to a control condition where the client has access to all preferred items and attention, and no instructions are placed. Elevated rates of target behavior in a test condition compared to the control are indicative of the functional reinforcer maintaining the target behavior.

After identifying the functional reinforcer, the therapist can rearrange environmental contingencies to encourage appropriate replacement behavior the individual can use to access the functional reinforcer. A function-based treatment for escape-maintained behavior may involve providing a break from non-preferred tasks contingent on the individual completing a small portion of the task and/or asking for a break (differential reinforcement) [2]. Extinction is also commonly employed, meaning the therapist no longer provides the functional reinforcer contingent on target behavior. For escape-maintained behavior, this means a break is not provided contingent on target behavior.

A review of published studies found that 32% of functional analyses identified target behavior maintained by negative reinforcement [4], with most functional analyses testing for target behavior maintained by escape from task instructions (e.g., academic tasks or chores) in the negative reinforcement condition without specifying the specific part of the instruction that evokes the behavior [4]. Thus, for a subset of these individuals, stimuli presented in conjunction with the instruction (e.g., vocal interaction, close proximity of an adult, and hand-over-hand prompting procedures) may be related to target behavior in addition to or in lieu of the presentation of the tasks itself (i.e., social avoidance) [5].

Contrary to assessment and treatment focused on target behavior maintained by escape from instructions [2], assessment and treatment research targeting escape from other aversive stimuli is less studied [6]. In one such study [5], the authors first demonstrated that target behavior for four individuals was maintained by social avoidance through functional analyses. The analyses included a social demand test condition where participants were exposed to vocal (continuous schedule) and physical (fixed time schedule) attention; contingent on target behavior, the therapist terminated social interaction for 30 s. Responding in this test condition was compared to a no-interaction control condition in a pairwise design.

Subsequently, researchers evaluated a series of treatment strategies [5]. Specifically, Harper et al. (2013) [5] started with a baseline condition identical to the social avoidance condition of the functional analysis described above, except that any appropriate interactions exhibited by the participants resulted in the delivery of a preferred edible item. This strategy (reinforcement of socially appropriate behavior with an edible) remained in place during subsequent treatment phases. Next, the researchers implemented vicarious reinforcement, where a confederate was present and interacted appropriately with the therapist. The therapist provided praise and an edible reinforcer to the confederate on a FT 30 s schedule for appropriate interactions. No social interaction was directed towards the participant, and target behavior was ignored. Next, the researchers started conditioning social interactions. In this phase, the therapist entered the room with preferred items and edibles, delivered an edible on a variable-time 15 s schedule, provided vocal and physical interactions every 30 s, and reinforced any target behavior with a 30 s break from interactions (no edible delivery during this time). The next treatment phase consisted of stimulus fading, where each component of social interaction (proximity, vocal attention, and physical attention) was systematically introduced in an a priori order hypothesized to be from least to most aversive (gradually increasing the time spent in vocal attention, increasing proximity to the participant, and increasing the time spent with physical attention). During stimulus fading, the participant had access to moderately preferred leisure items and target behavior was reinforced with a 30-s break from social interaction. If these treatment strategies were not successful, they initiated DRA with extinction. In this phase, the therapist provided praise, an edible reinforcer, and a break from the social interaction contingent on appropriate social interaction, and target behavior no longer resulted in a break. The authors found DRA with extinction was necessary in addition to the other strategies for reducing target behavior.

There are several potential explanations for this finding. First, it is possible that the delivery of the edible item was not a potent enough reinforcer or that the social conditioning phase was not implemented for long enough for counterconditioning to occur (i.e., for the social interaction to become less aversive). Second, it may be that stimulus fading was less effective because the types of social interaction were delivered in an order that experimenters determined was *likely* to be the least-to-most aversive. However, it is possible that this did not accurately reflect the preferences of the participants. That is, there may be idiosyncratic preferences across participants regarding the aversiveness of various forms of social interaction. One participant may find vocal interaction more aversive than physical interaction, while the opposite may be true for other individuals. It is possible that stimulus fading may be more effective if these idiosyncratic preferences are assessed and accounted for in treatment.

Slocum et al. (2022) [6] extended methods from Harper et al. (2013) [5] to assess these individual preferences. After completing a functional analysis to identify whether target behavior was maintained by social avoidance, Slocum et al. evaluated an assessment prior to treatment (i.e., negative reinforcement latency assessment, henceforth referred to as a social avoidance latency assessment (SALA)) to identify which forms of social interaction were most and least aversive. The SALA was based on previous demand latency assessments aimed at establishing hierarchies of task aversiveness such as schoolwork or daily living activities [7]. The SALA involved exposing participants to different forms of social interaction, terminating the social interaction contingent on target behavior, and measuring the latency from the introduction of the interaction to the first instance of target behavior (with shorter latencies indicating more aversive forms of interaction). This assessment identified a hierarchy of aversiveness to different social interactions among four participants [6]. Slocum and colleagues used the results of this assessment in stimulus fading to identify individualized hierarchies of aversiveness to different social interactions as opposed to a priori assumptions about the aversiveness employed by Harper et al.

Treatment components from Slocum et al. (2022) [6] were similar to Harper et al. (2013) [5] with a few modifications. First, Slocum and colleagues excluded vicarious reinforcement as this component was not successful as a standalone component for any participants in the prior study and requires the addition of a second therapist, which may limit generalizability to settings without this available resource. Second, Slocum and colleagues incorporated stimulus fading across all phases (compared to Harper and colleagues that included this as a distinct treatment phase separate from social conditioning and other treatment components). It is possible that social conditioning may be more successful if implemented in conjunction with stimulus fading as opposed to sequentially. Additionally, using stimulus fading throughout may minimize the aversiveness of baseline and treatment phases for participants. Despite the inclusion of the SALA and these modifications, DRA with extinction was ultimately needed for the participant to reduce target behavior. A third modification made by Slocum and colleagues was to reinforce a communication response as the alternative response in DRA rather than appropriate social interaction. Given the single participant in the research by Slocum et al., replication is needed to evaluate the impact of an SALA on assessment and the treatment results for target behavior maintained by social avoidance. Replication will allow for an evaluation on whether stimulus fading is more successful when introductions to different social interactions are carried out based on individual preferences.

Even if differential reinforcement is eventually needed, there may still be benefit in the inclusion of individualized stimulus fading in the treatment package. These strategies allow for the start of treatment with desensitization to social interaction using the least aversive form for that specific individual. Thus, even if differential reinforcement is ultimately needed, stimulus fading and social conditioning can be used to minimize the aversiveness of stimuli presented in treatment, likely improving the social validity of treatment.

The current paper has three primary aims. First, given the limited demonstrations of functional analyses including social avoidance test conditions, we aim to replicate the past literature (e.g., [5]) through assessing for a social avoidance function with five participants for whom a social avoidance function was hypothesized based on previous assessment results or parent reports. Second, we replicate findings from Slocum et al. (2022) [6] by implementing the social avoidance latency assessment with all participants to identify a hierarchy of aversiveness of social interaction. Last, we replicate Slocum and colleagues’ intervention strategies with additional participants.

## 2. General Method

### 2.1. Participants and Setting

Five individuals attending an intensive outpatient program for the assessment and treatment of aggression, self-injury, property destruction, and/or elopement participated. The mean age was 16.6 years (range 13–19). Most participants (four) were male and one was female; three participants were White and two were Black. All participants had a diagnosis of autism spectrum disorder and an intellectual disability. Information on verbal abilities was also gathered from a record review. Vincent used gestures to communicate primarily but also used one- and two-word phrases in both English and Russian. Chris, Kelsey, Miles and Mike used gestures, signs, and pictures to communicate in English. Participants were included if functional analysis results revealed a social avoidance function and the direct clinical supervisor wanted to conduct the current protocol. There was not a formal requirement that the case management team had to follow the current protocol, but during the time this study was conducted, most of the management team supported this protocol. All sessions were held in a room with padded walls and a one-way mirror. At least one therapist was present in the room, and at least one therapist collected data behind the one-way mirror. Therapists were Registered Behavior Technicians within our clinical program trained to reliability. Furniture varied across participants but included either a table and chairs or a beanbag.

### 2.2. Ethical Considerations

All participants completed study procedures as part of their clinical admission in a treatment program. The clinicians responsible for the cases agreed all procedures were in the best interest of the child’s clinical progress to achieve the treatment goals agreed on by the caregivers at the start of the admission. Clinicians met with caregivers weekly to discuss assessment and treatment procedures and outcomes. Consent was obtained by legal guardians of all participants for clinical care. After completion of the clinical admissions and data collection, this study was approved as a retrospective chart review by the first author’s second affiliation. Consent and assent were waived by the IRB given the retrospective nature of this study and due to the minimal risk associated with this study. No new procedures were implemented with the participants for this study.

### 2.3. Responses and Interobserver Agreement

In the functional analysis, baseline, and treatment sessions, target behavior was scored as a frequency measure. Three participants’ data were collected using paper/pencil (Miles, Vincent and Chris) and two participants’ data were collected using a computer-based system (Kelsey and Mike). Target behavior and mands were defined on an individual basis (see Table 1 with more complete behavioral definitions available from the first author upon request). The frequency of target behavior was converted into a rate (responses per minute) for functional analysis, baseline and treatment conditions. In the social avoidance latency assessment, the latency in seconds from the start of the session (defined by the therapist as counting down from three and then saying “start”, which was immediately followed by initiating the social interaction) to the onset of the first instance of target behavior was recorded. If no target behavior occurred, the maximum session duration was recorded (e.g., 300 s for a 5 min session).

A second independent observer collected interobserver agreement (IOA) across a subset of sessions in all conditions. For paper/pencil data collection for frequency measures and all latency measures, total IOA was calculated by taking the observer’s record with the smaller recorded frequency or latency divided by the larger and multiplying by 100 to produce a percentage agreement. For rate measures for computer-based data collection, 60 s of partial interval IOA was calculated by taking the smaller value divided by the larger value and multiplying by 100 for each 60 s session interval and averaging across the full session.

For the functional analysis, two observers collected data for 37%, 58%, 25%, 26%, and 80% of sessions for Kelsey, Mike, Miles, Vincent and Chris, respectively. IOA averaged 95% (range = 77–100%) for Kelsey, 97% (range = 92–100%) for Mike, 100% for Miles, 98% (range = 79–100%) for Vincent, and 100% for Chris. For the social avoidance latency assessment, two observers collected data for 27%, 30%, 25% 30% and 33% of sessions for Kelsey, Mike, Miles, Vincent and Chris, respectively. IOA averaged 93% (range = 64–99%) for Kelsey, 95% (range = 86–100%) for Mike, 90% (range = 75–100%) for Miles, 100% for Vincent, and 99% (range = 95–100%) for Chris. For treatment sessions, two observers collected data for 39%, 45%, 26%, 37% and 29% of sessions for Kelsey, Mike, Miles, Vincent and Chris, respectively. IOA averaged 97% (range = 79–100%) for Kelsey, 96% (range = 60–100%) for Mike, 99% (range = 80–100%) for Miles, 98% (range = 67–100%) for Vincent, and 97% (range = 50–100%) for Chris.

### 2.4. Functional Analysis

Preference assessments informed preferred items for tangible and toy play conditions of the functional analysis. Paired stimulus preference assessments were carried out with four participants (Vincent, Chris, Kelsey, and Mike) [8], free operant preference assessments for two participants (Chris and Kelsey) [9], and multiple stimulus without replacement assessments for two participants (Kelsey and Miles) [10].) Therapists implemented more than one preference assessment if the initial assessment did not provide a clear hierarchy of preferred items.

Therapists implemented functional analyses for all five participants. All sessions were 10 min. The assessment consisted of a combination of toy play (i.e., control), attention, tangible, escape from task instruction, social avoidance, and no-interaction conditions. Procedures were similar to previous research [1] with some modifications as described in past research [6,11]. In the social avoidance condition for Mike and Miles only, vocal attention was delivered. For the remaining participants (Kelsey, Vincent and Chris), both vocal and physical attention was delivered. The type of social attention was delivered continuously. Contingent on target behavior, the therapist stopped the social interaction for 30 s. The type of social interaction delivered during these sessions was determined during caregiver interviews to be as similar as possible to what type of social interaction the caregiver would provide.

Vincent’s functional analysis started with a multielement design with escape from task instruction, social avoidance, tangible, and toy play conditions, followed by a reversal to further examine social avoidance and tangible conditions. In the reversal, due to lower and more variable levels of target behavior, we conducted an additional reversal phase. For Chris, a multielement design functional analysis included attention, tangible, social avoidance, toy play, and no-interaction conditions. A reversal design was used for the remaining participants evaluating no interaction, social avoidance, demand, and tangible conditions for Kelsey; and social avoidance and no-interaction conditions for Mike.

### 2.5. Latency Assessment

In the social avoidance latency assessment (SALA) [6], various types of social interaction were presented, one per session, to determine a hierarchy of aversiveness. The types of social interaction delivered in the latency assessment varied for participants (see Table 2) and were selected based on parent reports and therapist observations of the types of interaction that may evoke target behavior, with all types of interaction representative of what a child may encounter in their daily lives at home or school. During the SALA, the session duration was either 5 (Kelsey and Mike) or 10 min (Vincent, Chris, and Miles). Session duration varied across participants because of clinical judgement. For example, shorter sessions (5 min) were used if previous observations suggested target behavior would likely be captured in this time frame. At session onset, the therapist initiated the interaction and provided it continuously. Contingent on target behavior, the therapist terminated social interaction. Across all participants, a no-interaction control condition was also included where the therapist did not interact with the participant for the duration of the session. The type of interaction presented in each session was randomized without replacement for all participants except Miles, so all social interaction types were presented once in a series of sessions. Three to five series were implemented (i.e., each form of interaction was evaluated at least three times). For Miles, a reversal latency assessment was used due to a history of variability in responding in previous assessments.

For all participants, latency to target behavior was measured and averaged for each social interaction condition. For Vincent, Kelsey, and Mike, the latency assessments followed the functional analysis to inform treatment. Results were used to determine the presentation sequence of social interaction in stimulus fading in treatment (described in detail below). For Chris and Miles, the latency assessments preceded the functional analysis described above, and results were used to inform both the functional analysis and treatment. The order of the latency assessments was selected by the case manager in charge of the client’s clinical intervention.

### 2.6. Treatment

#### 2.6.1. General Procedure

All sessions were 10 min. Across baseline and the treatment sessions described below, stimuli necessary for the target mand (card or button similar to a BIGmack) was available based on the target mand modality for each participant.

#### 2.6.2. General Baseline Contingencies

In baseline, the selected social interaction was presented at the onset of the session. Contingent on target behavior, the therapist terminated the social interaction and moved to the other side of the room or left the room for 30–120 s (depending on the participant; see Table 3). The protocol included leaving the room if the clinical team decided (based on previous observations) that moving to the other side of the room was not a sufficiently potent negative reinforcer. Contingent on a target mand, an edible was delivered; however, the functional reinforcer (escape) was not. Note that stimulus fading (described below) was initiated from the start of baseline. That is, we gradually faded in social interaction types based on aversiveness under baseline continencies. Contingent on elevated target behavior, we then initiated social conditioning (also described below) while remaining in the baseline contingencies of reinforcing target behavior with escape from social interaction. This is identical to the contingencies in the social conditioning phase from Harper and colleagues.

**Stimulus Fading in Baseline**. Within baseline contingencies, the therapist began by engaging in the least aversive form of social interaction (determined by the SALA). We moved to the next most aversive form of social interaction as low levels of target behavior were observed, defined as at least two consecutive sessions with less than 0.3 RPM of target behavior. Occasionally more stringent criteria were set (e.g., more sessions required) depending on visual analysis of the data and based on trends and previous variability observed for each participant. Once a form of social interaction evoked target behavior in the baseline, that type of interaction was maintained in the subsequent phases described below. That is, when we moved between phases, we did not start over with stimulus fading. Stimulus fading was not carried out with Kelsey as the clinician managing her case felt it was unlikely to be successful without additional components. Instead, Kelsey began in social conditioning described below.

**Social Conditioning in Baseline**. Social conditioning included pairing session contingencies, and thus various forms of social interaction, with preferred items previously established through preference assessments. For all participants, edibles were delivered contingent on a target mand as well as on a fixed time schedule (similar to Harper et al. (2013) [5]). Leisure items were also used for some participants. The components included in social conditioning varied across participants based on participants’ preferences of various edible and leisure items (see Table 3). Target behavior continued to result in the removal of social interaction (reinforcement), aligned with procedures by Harper and colleagues.

#### 2.6.3. Differential Reinforcement of Alternative Behavior (DRA) with Extinction (EXT)

In the DRA+EXT phase, contingencies for target behavior and target mands were reversed. Target mands produced 30–120 s of escape from social interaction. The reinforcement interval varied across participants; however, the reinforcement interval for mands in treatment was the same interval used for target behavior in baseline. Target behavior resulted in the continued presentation of social interaction (i.e., social avoidance extinction). Treatment components related to social conditioning (e.g., delivery of an edible and leisure items on a fixed time schedule) were no longer in place. Stimulus fading continued in this phase. DRA with extinction was initially implemented with the level of social interaction from the previous phase, and for some participants, stimulus fading continued within the DRA-plus-extinction phase.

## 3. Results

**Figure 1:**
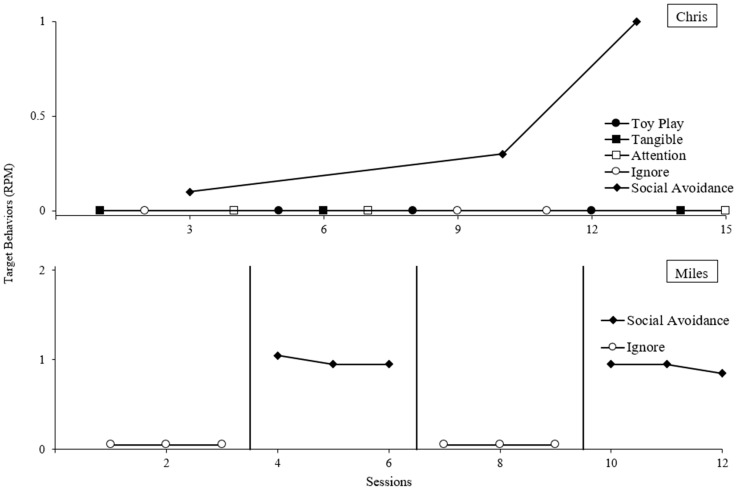
Functional analysis results for Chris and Miles.

**Figure 2:**
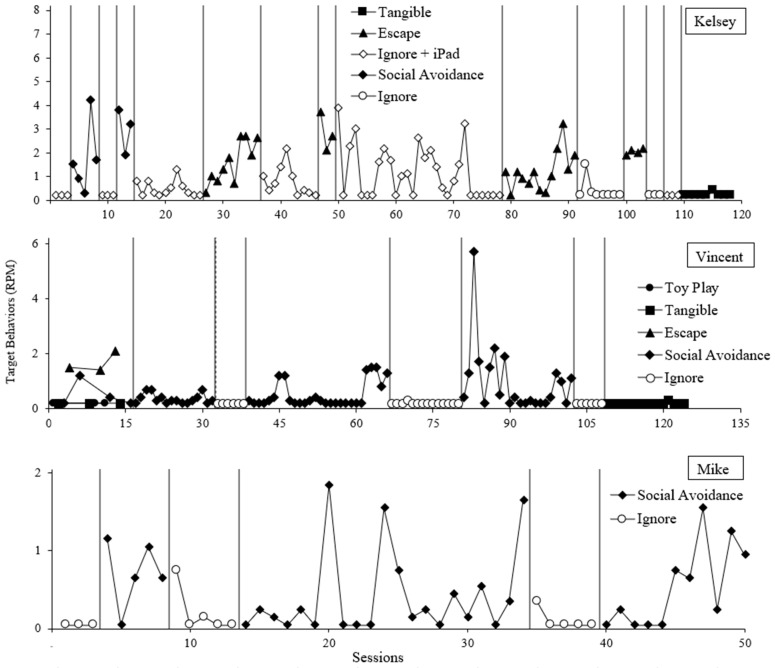
Functional analysis results for Kelsey, Vincent, and Mike.

For all participants, elevated rates of target behavior occurred during the social avoidance condition compared to the control in the functional analyses (Figure 1 and Figure 2). Kelsey and Vincent also displayed target behavior in the escape from task instructions condition.

Following Kelsey’s reversal between the ignore + iPad condition and the social avoidance condition, some unexplained variability emerged in the ignore + iPad condition when used as the control for the escape condition. We then elected to use an ignore condition as the control for the escape test. Given the clear differentiation between the initial reversal for social avoidance, the low latencies of target behavior in the SALA for many attention types, and Kelsey engaging in target behavior during baseline of the treatment evaluation, there is clear evidence of a social avoidance function.

Variability also occurred with Vincent’s social avoidance condition. It appears the established operation for negative reinforcement from social interaction was fleeting, resulting in long stretches without target behavior followed by several sessions with high rates of target behavior in the social avoidance condition. However, given the immediate and sustained low rates of target behavior in control (with phase duration comparable to the stretches of low rates in the social avoidance condition), a social avoidance function was concluded.

**Figure 3:**
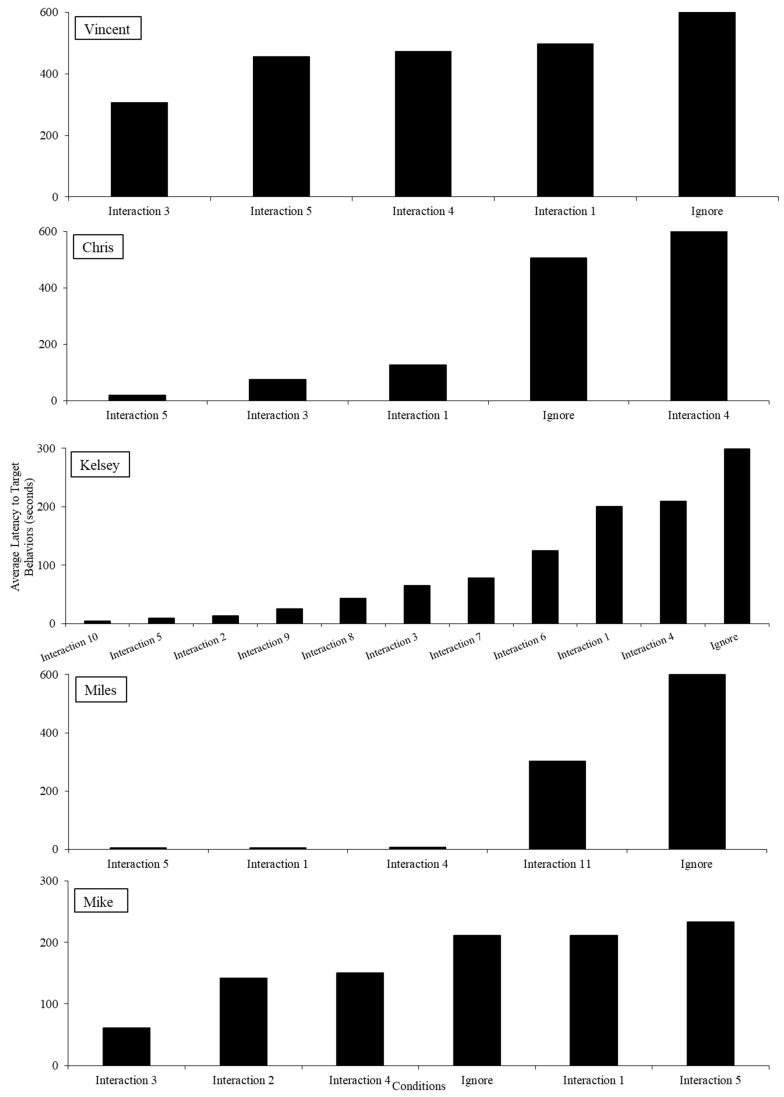
Social avoidance latency assessment results.

In the SALAs, hierarchies in latency to target behavior can be observed (Figure 3). For all participants, physical touch evoked challenging behavior quickly with longer latencies in the control condition. Variability across the other conditions (e.g., physical proximity and vocal interactions) was noted, representing idiosyncratic findings across participants.

**Figure 4:**
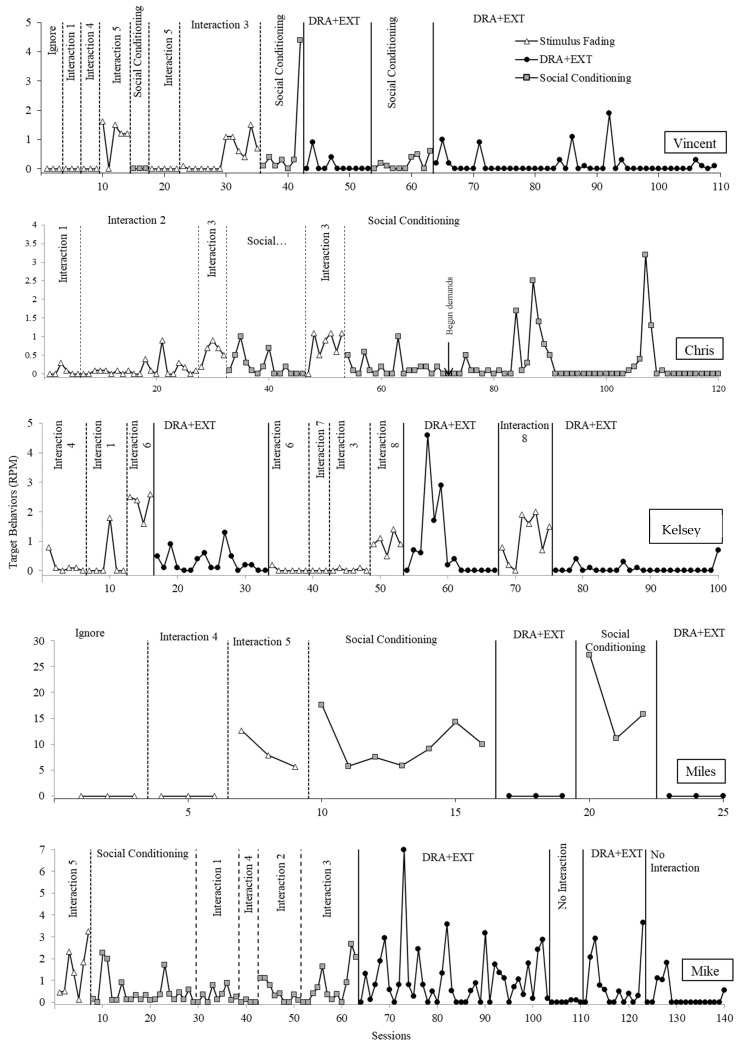
Treatment evaluation results.

Figure 4 displays the rate of target behavior for all participants in treatment. For Vincent, we began with the control context of the latency assessment, which was the condition producing the longest latency to target behavior. Following low levels of target behavior, we moved up the hierarchy of aversive contexts until the fourth most aversive form of social interaction (physical attention) reliably evoked target behavior. After adding in social conditioning under baseline contingencies, we observed a reduction in target behavior. Therefore, we continued to move up in the hierarchy of aversive social interaction contexts. Physical and vocal attention combined from the therapist (the fifth most aversive) began to evoke target behavior. Therefore, we moved to the DRA+EXT phase, which resulted in a suppression in target behavior. Thus, we were able to progress through five different social interaction types that evoked targeted behavior in the latency assessment in the stimulus fading and social conditioning conditions before DRA+EXT was needed. Finally, we reversed back to social conditioning and then returned to DRA+EXT to demonstrate experimental control.

Chris’s data are displayed in the second panel of Figure 4. We did not implement baseline contingences with the two types of social interaction that were least aversive as no target behavior was observed during all but one of these sessions of the social avoidance latency assessment. We instead started with a form of social interaction that evoked target behavior consistently, and we observed low rates of target behavior in baseline. Target behavior remained low until the third most aversive form of social interaction (physical and vocal). When social conditioning was implemented with physical and vocal attention, target behavior, while variable, was overall lower than the prior phase with several sessions without target behavior. We then reversed and replicated the effect. DRA+EXT was not needed for Chris, and we stopped our evaluation without moving to subsequent aversive forms of social interaction as the case manager decided that tolerating that level of interaction was sufficient for generalization.

For Kelsey, low rates of target behavior were observed when the two least aversive forms of social interaction were implemented. Target behavior emerged during the third most aversive form of social interaction (questions). Thus, we were able to progress through two different forms of social interaction in the stimulus fading phase before moving to the DRA+EXT phase. (Kelsey’s case manager elected not to implement the social conditioning baseline phase.) However, upon introduction of the DRA+EXT phase, target behavior reduced to low levels. Upon a return to baseline to establish experimental control, target behavior remained at low levels until it re-emerged during the sixth most aversive form of social interaction. Thus, we were able to progress through three additional forms of social interaction before DRX+EXT. The DRA+EXT phase was again implemented and resulted in low rates of target behavior. Upon return to baseline, target behavior was again observed at higher levels which returned to near zero levels when DRA+EXT was reintroduced.

For Miles, there was a protocol error, and the therapist skipped the least most aversive form of social interaction and instead began with the second least aversive form. Target behavior emerged upon introduction of the third most aversive form of social interaction, and introduction of the social conditioning phase did not result in a reduction in target behavior. Similar to Kelsey, the DRA+EXT phase resulted in suppression of target behavior. A return to social conditioning resulted in a re-emergence of target behavior followed by suppression again in the DRA+EXT phase.

We were unable to demonstrate a clear treatment effect with Mike. We observed elevated rates of target behavior in the first social interaction baseline condition. We then progressed from less to more aversive types of social interaction with variable but low (compared to baseline) rates of target behavior in the social conditioning condition. When we included the fifth most aversive type of attention, we saw an increase in target behavior and moved to the DRA+EXT phase. Thus, we were able to progress through four types of social interaction before the DRA+EXT phase. However, this treatment condition was unsuccessful at reducing target behavior. Next, we elected to move to a no-interaction context to demonstrate that social interaction was the cause of elevated target behavior. Following low levels of target behavior, we reversed between our intended intervention and a no-interaction context, demonstrating that treatment was ineffective.

## 4. Discussion

Escape from social interaction, or social avoidance, is an under-studied function for target behavior in the behavior-analytic literature. This is especially problematic given how commonplace social interaction is in everyday life. Individuals who engage in target behavior to escape the presence of others or other social interaction may warrant additional preliminary intervention before introducing instructions in treatment.

The current study provides an important contribution to the literature on target behavior maintained by social avoidance. First, it replicates functional analysis conditions designed to identify target behavior maintained by social avoidance [5,6]. Compared to other functions of target behavior, this type of condition has been presented very few times in the past literature, thus replication is meaningful. Additionally, we replicated the findings of [6] indicating the success of the latency assessment in identifying a hierarchy of aversiveness of social interaction. Given the potential of this assessment to personalize the types of interaction used in assessment and treatment, this is an important replication that sets the ground for the use of this assessment in clinical practice and future research. Last, our results provide an important extension of the past literature by using the latency assessment to guide treatment procedures presented in past studies [5], allowing for a more individualized and personalized approach. In doing so, we present a framework for the treatment of target behavior maintained by social avoidance that was effective for four out of five participants.

The inclusion of a latency-based assessment to inform stimulus fading permitted what is likely a more socially valid approach than previous work by gradually introducing social interaction, starting with those that are least aversive from the onset of the treatment evaluation (i.e., in baseline). Often when clinicians implement baseline contexts for targeted behavior maintained by negative reinforcement, they begin with the most aversive evocative event to reduce the chance of a false-negative function (i.e., reduce the chance of not finding a negative reinforcement function that is present in the natural environment). However, this approach results in the repeated presentation of an aversive context, which may have long-term detrimental effects, such as the therapist being socially conditioned as aversive and potentially creating an overall aversive experience for the client or participant. Gradually introducing potentially aversive situations from least to most aversive provides an alternative approach that may be more preferred for participants or clients while maintaining a reduced chance of a false negative (failing to observe target behavior) by gradually increasing the aversiveness until target behavior is observed.

Second, this study included stimulus fading and social conditioning components that are often missing from past studies targeting behavior maintained by negative reinforcement focused on the use of operant contingencies [12,13]. While DRA and extinction were ultimately needed for most participants, we were able to progress through some of the social interaction while maintaining low rates of target behavior with social conditioning alone for four participants (Vincent, Chris, Kelsey and Mike). This is important as it is aligned with the ultimate goal of treatment. That is, it is ideal in treatment to not only teach an alternative response (e.g., mand) to access social avoidance, but to also disassociate therapists or instructors in the environment as aversive stimuli altogether. More research is needed to determine the mechanism responsible for the effectiveness of social conditioning for some participants. While DRA+EXT was needed for most participants, the fact that some progress was made in terms of tolerating aversive forms of social interaction without it suggests that individualizing stimulus fading in the context of social conditioning can still be beneficial. Future evaluation is needed to replicate this finding and to find additional ways to maximize the efficacy of these treatment strategies without requiring DRA+EXT. For example, it is possible that introducing more intermediate steps (e.g., fading in more aversive interactions based on time) would allow for further success without the use of DRA and extinction. We also evaluated combined topographies of target behavior in assessment and treatment. Is it possible that different topographies were maintained by different functional reinforcers and treatment evaluations may have been more sensitive for certain topographies? Future research should evaluate the success of treatment components with this consideration.

The current study has several limitations that warrant discussion. First, the order of assessments was inconsistent across participants. Some participants were exposed to the functional analysis first and others were exposed to the SALA first. It is unclear how these variations impact findings. Additionally, if the SALA precedes the functional analysis, future research should evaluate using the results to personalize the type of interactions included in the functional analysis. In general, because we conducted this evaluation as part of ongoing clinical services, more of a systematic approach to identifying participants (i.e., including all participants with a social avoidance function) or decreasing some inconsistencies would have improved the internal validity of this study.

Other limitations include differences across participants in how social conditioning components were implemented, whether they were implemented from the start of baseline or as a separate phase, and whether all conditions from the SALA were used in stimulus fading. All participants received an edible contingent on target mands (which was similar to procedures from Harper et al. (2013) [5]), but other social conditioning components varied across participants. This was carried out based on preferences demonstrated by participants in assessments, observations prior to the functional analysis, and interviews with parents. However, it is not clear if these differences impacted results. Variations in conditions included in treatment (e.g., Chris skipping conditions that did not evoke target behavior in the SALA) were made by the treatment team with the goal of expediting treatment. However, future research should evaluate if this decision differentially impacts outcomes. We also did not collect sufficient data to report on rates of target mands; future research should consider this variable.

Another limitation is the lack of a robust effect regarding Vincent’s reversal to social conditioning. In the third social conditioning phase (see sessions 54–63), there is an increase in target behavior; however, these are lower levels when compared to baseline. One likely contributing factor here is that we used two antecedent manipulations even in baseline (stimulus fading and social conditioning), so more robust reversals may not be predicted. Our interobserver agreement calculations for the paper/pencil data collection may also pose a limitation as the full session calculations are not sensitive to disagreements when a specific behavior is scored. Future research should use data collection methods that are more robust.

Finally, the current evaluation used a continuous reinforcement schedule (FR1) for target mands. Many empirical evaluations involving functional communication for other maintaining variables of target behavior include tactics such as multiple schedules [14] or delay/denial training [15]. These procedures should be applied to social avoidance to demonstrate ways of making the intervention more feasible long term. Additionally, given our limited number of participants, all findings (including whether the SALA will identify a hierarchy of aversiveness in social interaction) require additional replication.

Functional analysis technology does not limit us to the examination of the common maintaining reinforcers for target behavior [16]. Although many individuals engage in target behavior to access attention, preferred items or activities, or to escape some form of instruction, other more idiosyncratic maintaining variables should be considered. As a field, it is critical we continue to expand the use of experimental analyses and subsequent function-based treatments to ensure we are implementing a comprehensive intervention.

Future research should evaluate when a social avoidance condition should be included in functional analyses. Past research suggests elevated rates of target behavior in a toy play context may be predictive of a social avoidance function. However, this may not always be the case (e.g., Chris) for several reasons. First, many researchers implement a toy play context where interaction occurs on a fixed time schedule (e.g., FT 30 s). This may not arrange a strong enough established operation for target behavior maintained by social avoidance. Second, many researchers include highly preferred leisure items in the toy play condition which may compete with social avoidance reinforcement. Finally, repeated presentation of social interaction without the reinforcer of escape in control conditions may result in extinction. Future research should focus on identifying screeners for when social avoidance test conditions should be included in functional analyses so the field can have a better idea of the prevalence of this function.

## Figures and Tables

**Table 1:** Participant demographics, target behavior, and target mands.

Participant	Gender	Age	Diagnosis	Intellectual Disability	Target Behavior	Target Mand
Vincent	Male	19	ASD	Moderate	AGG, DIS, ELOPE	Vocal/button press
Chris	Male	13	ASD	Mild	AGG, DIS, SIB	Button press
Kelsey	Female	18	ASD	Mild	AGG, DIS, SIB	Vocal/card exchange
Miles	Male	18	ASD	Unspecified	AGG, DIS, SIB	Card exchange
Mike	Male	15	ASD	Severe	AGG, SIB	Button press

*Note*. AGG = aggression, DIS = disruption (i.e., behavior that could result in damage to items, walls, or furniture), SIB = self-injurious behavior, ELOPE = elopement.

**Table 2:** Types of interactions during assessment and treatment.

Interaction #	Type of Social Interaction	Definitions of Social Interaction
1	Close proximity	Therapist within 3 feet of client
2	Close proximity + vocal	Therapist within 3 feet of client and talking
3	Physical + vocal	Physical touch and talking
4	Vocal	Talking (not within 3 feet of client)
5	Physical attention	Physical touch
6	Questions	Therapist asking questions
7	Noise	Therapist making sounds
8	High fives	Therapist giving high fives
9	Close proximity + questions	Therapist within 3 feet of client and asking questions
10	Close proximity + noise	Therapist within 3 feet of client and making sounds
11	Proximity + no movement	Therapist within 3 feet of client and no movement

*Note*: Interaction # corresponds to numbers in Figures 3 and 4. For interaction #11, the therapist began the session within 3 ft of the client; however, the therapist did not move closer to the client if the client moved around the room. For other conditions with close proximity, the therapist continued moving to be within 3 ft of the client at all times.

**Table 3:** Baseline (including reinforcement duration and social conditioning) components per participant.

Participant	Baseline (and Treatment) Rein Duration	Rein Position Other Side/ Leave Room	Edible for Manding	Leisure Item Delivered (Soc Cond Phase)	Fixed-Time Edible Delivery (Soc Cond Phase)
Kelsey	30 s	Other Side	+	−	−
Mike	120 s	Leave room	+	+	+ (FT 2 min)
Miles	120 s	Leave Room	+	−	+ (FT 2 min)
Vincent	60 s	Other side	+	+	−
Chris	120 s	Leave room	+	+	+ (FT 5 min)

*Note*. Included components are denoted by a +. Excluded components are denoted by a −. FT = fixed time.

## Data Availability

Data available upon request from the corresponding author.
